# Red blood cells stabilize flow in brain microvascular networks

**DOI:** 10.1371/journal.pcbi.1007231

**Published:** 2019-08-30

**Authors:** Franca Schmid, Matthew J. P. Barrett, Dominik Obrist, Bruno Weber, Patrick Jenny

**Affiliations:** 1 Institute of Fluid Dynamics, ETH Zurich, Sonneggstrasse 3, Zurich, Switzerland; 2 Institute of Pharmacology and Toxicology, University of Zurich, Winterthurerstrasse 190, Zurich, Switzerland; 3 Neuroscience Center Zurich, University and ETH Zurich, Winterthurerstrasse 190, Zurich, Switzerland; 4 ARTORG Center for Biomedical Engineering Research, University of Bern, Murtenstrasse 50, Bern, Switzerland; University of California at San Diego, UNITED STATES

## Abstract

Capillaries are the prime location for oxygen and nutrient exchange in all tissues. Despite their fundamental role, our knowledge of perfusion and flow regulation in cortical capillary beds is still limited. Here, we use *in vivo* measurements and blood flow simulations in anatomically accurate microvascular network to investigate the impact of red blood cells (RBCs) on microvascular flow. Based on these *in vivo* and *in silico* experiments, we show that the impact of RBCs leads to a bias toward equating the values of the outflow velocities at divergent capillary bifurcations, for which we coin the term “*well-balanced bifurcations*”. Our simulation results further reveal that hematocrit heterogeneity is directly caused by the RBC dynamics, i.e. by their unequal partitioning at bifurcations and their effect on vessel resistance. These results provide the first *in vivo* evidence of the impact of RBC dynamics on the flow field in the cortical microvasculature. By structural and functional analyses of our blood flow simulations we show that capillary diameter changes locally alter flow and RBC distribution. A dilation of 10% along a vessel length of 100 μm increases the flow on average by 21% in the dilated vessel downstream a *well-balanced bifurcation*. The number of RBCs rises on average by 27%. Importantly, RBC up-regulation proves to be more effective the more balanced the outflow velocities at the upstream bifurcation are. Taken together, we conclude that diameter changes at capillary level bear potential to locally change the flow field and the RBC distribution. Moreover, our results suggest that the balancing of outflow velocities contributes to the robustness of perfusion. Based on our *in silico* results, we anticipate that the bi-phasic nature of blood and small-scale regulations are essential for a well-adjusted oxygen and energy substrate supply.

## Author summary

Glucose and oxygen are key energy sources of the brain. As energy storage capabilities are limited in the brain, a continuous supply of oxygen and glucose via the bloodstream is crucial for the brain’s functioning. The bulk of discharge occurs at the level of capillaries, which are the smallest and most frequent vessels of the cortical vasculature. Nonetheless, our understanding of perfusion and topology of the capillary bed is still limited. Here, we use *in vivo* two-photon based blood flow measurements and numerical simulations in large realistic microvascular networks to study the flow in the cortical microvasculature. Our results reveal that the impact of red blood cells enhances the robustness of microvascular perfusion and increases the heterogeneity in red blood cell distribution. It is well established that higher neuronal activity leads to an increase in blood flow. However, the precise regulation mechanisms and their spatial extent remain largely unknown. We show that small-scale regulations locally alter flow and red blood cell distribution. We suggest that these mechanisms are key for an efficient and flexible circulatory system. Moreover, our results reveal a novel role of the bi-phasic nature of blood.

## Introduction

Tissues depend on a continuous supply of oxygen and energy substrates delivered via the bloodstream. This is particularly evident in the brain, which contains limited energy reserves and undergoes rapid and substantial increases in blood flow during neuronal activation (*neurovascular coupling*) [1].

Robustness of perfusion is crucial to guarantee a sustained nutrient supply throughout the tissue. Occlusion experiments have been used to study robustness of perfusion [2–6]. Their results suggest that single vessel occlusion at the pial and capillary level does not lead to a complete cessation of flow but to a redistribution of flow. Indeed, for capillaries, three branches downstream from the site of occlusion the red blood cell (RBC) flux recovers to 45% of its baseline value [5]. Occlusions of penetrating vessels prove to be more severe [5, 6]. However, these studies focus on the effect of the occlusion and it remains largely unknown if the observed flow redistribution results exclusively from the vascular topology, or if further mechanisms are relevant.

Up-regulation of flow in response to neuronal activation is another key feature of the cortical blood supply. The increase in flow rate results from vasodilations of arterioles and capillaries [7–11]. It is still matter of on-going debate if dilations at capillary level are active or passive [10–12]. Either way, capillaries are the ideal location for nutrient exchange and the most abundant vessel type [13]. Those aspects underline that the perfusion of the capillary bed is at the very basis of nutrient supply in all tissues. Nonetheless, many open questions regarding capillary perfusion patterns during baseline and activation remain to be answered.

Various experimental and numerical studies show that the perfusion of the capillary bed is highly heterogeneous [12, 14–23]. This heterogeneity is evident for multiple perfusion characteristics, e.g. hematocrit and velocity distribution [14–17, 23] as well as capillary transit time (CTT) and capillary outlet saturation [18, 24–26]. These characteristics are relevant for oxygen and nutrient supply [15, 18, 19, 26], and are also linked to diseases and aging [24, 27, 28]. For example it has been shown that CTT homogenizes during activation [18, 19, 29] and that this is beneficial for oxygen extraction [24]. However, in a mouse model of Alzheimer’s disease CTT homogenizes less during activation than in wild type mice [27].

Little is known about the mechanisms leading to the homogenization of capillary flow during activation. As the homogenization is initiated before the up-regulation of flow [19] it seems likely that dilations and constrictions at the level of individual capillaries are relevant. However, *in vivo* such alterations are difficult to measure [11] and quantitative numerical investigations are lacking.

Here, we use numerical simulations in anatomically accurate microvascular networks (MVNs) [6, 12] and *in vivo* measurements to quantitatively describe and analyse the flow field in the capillary bed. In further structural and functional analyses we investigate the changes resulting from the dilation of individual capillaries and quantify the impact of single capillary dilation. Capillary dilation is also used as an example scenario to study robustness of perfusion in response to local alterations.

In all investigations we highlight the impact of the bi-phasic nature of blood (plasma and RBCs) [23, 30–34]. This is motivated by the significant impact of RBCs on the microvascular flow field. The term “impact of RBCs” refers to the effects due to the presence of RBCs in comparison to pure plasma flow without RBCs. On the scale of MVNs these effects become apparent in a threefold manner: (1) a general increase in flow resistance [35], (2) the non-homogeneous distribution of RBCs [17, 20, 23, 36–38] and (3) the altered flow and pressure field due to the non-homogeneous RBC distribution [32]. All three effects are direct consequences of the RBC dynamics in microvascular flow (Fahraeus-Lindqvist effect and phase-separation effect, Methods).

So far, most studies addressing the impact of RBCs have been performed in the vasculature of the muscle or the mesentery, and the analysed vascular networks were at maximum 1,000 vessels in size [23, 32–34, 39–43]. These studies show that the particulate nature of blood induces temporal fluctuations in the flow field. Moreover, they indicate that phase-separation is the key mechanism leading to hematocrit heterogeneity. A recent work performing direct numerical simulations of bi-phasic blood flow in MVNs with ~50 vessels provides novel evidence that these effects are most pronounced at capillary level [23].

It has been shown, that the continuum approach to model the RBC-phase [41] tends to under predict the impact of RBCs [32, 34]. Therefore, we believe that the tracking of individual RBCs is crucial to accurately model and study the impact of RBCs. This becomes even more relevant for large realistic MVNs, where the capillary bed forms an interconnected mesh-like structure [6] with on average six capillary segments between arteriole and venule [44]. However, most numerical models working with large MVNs do not track individual RBCs [20, 36, 41, 45]. Thus, to the best of our knowledge this is the first study investigating the impact of RBCs in cortical MVNs with more than 10,000 vessels. In contrast to previous works, our focus is on quantitatively describing the impact of RBCs across scales, i.e. from the very local impact at individual bifurcations to the large-scale impact across the entire MVN. Our local investigations are complemented by *in vivo* measurements at individual cortical capillary bifurcations. Additionally, we studied the role of RBCs during capillary diameter changes and for robustness of perfusion. We also comment on capillary dilation as a candidate mechanism for small-scale regulations.

## Results

### *In vivo* measurements of outflow velocities at capillary bifurcations

**Fig 1:**
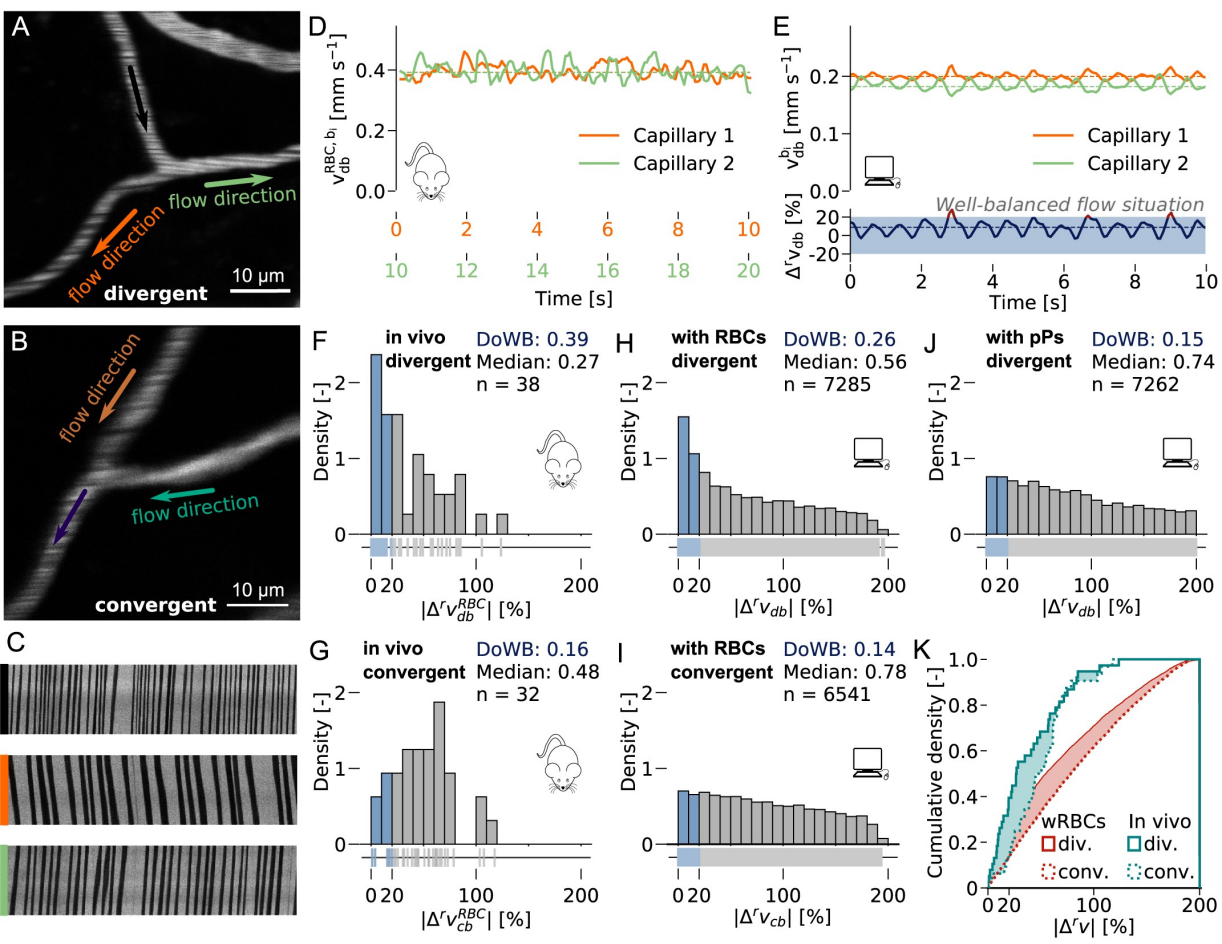
The relative velocity difference is smaller at divergent than at convergent capillary bifurcations. (**A-B**) Examples of an *in vivo* divergent (A) and convergent (B) capillary bifurcation. (**C**) Line scans for the divergent bifurcation in (A). (**D**) RBC velocity $v_{db}^{RBC,b_{i}}$ in the daughter vessels of a divergent bifurcation *in vivo*. Dotted lines: median of each velocity. The velocity measurements have been performed consecutively (Methods). Further examples: S5 Fig. (**E**) Upper plot: Bulk flow velocity $v_{db}^{b_{i}}$ in the daughter vessels of a divergent bifurcation for the simulation with red blood cells (RBCs, Methods). The time course has been smoothed by a moving average (Methods). Lower plot: Instantaneous relative velocity difference $\Delta^{r}v_{db}$. Dotted lines: median of each variable. Blue box: $|\Delta^{r}v_{db}|\leq 20\%$, *well-balanced flow situation*. Further examples: S6 Fig. (**F-J**) Histogram: Distribution of the relative velocity difference at divergent $\left|\Delta^{r}v_{db}\right|$ (F, H, J) and convergent $\left|\Delta^{r}v_{cb}\right|$ (G, I) capillary bifurcations for the simulation with RBCs (H, I) with passive particles, pPs (J) and *in vivo* (superscript: RBC; F, G). The histograms are normalized, i.e., the density of the underlying empirical distributions is displayed. Lower plot: Raw data for histograms. Blue bars: *Well-balanced bifurcations* ($|\Delta^{r}v_{db}|\leq 20\%$). DoWB: Degree of *well-balanced bifurcations*: ratio of the number of *well-balanced bifurcations* to the total number of bifurcations (n). A detailed analysis of the distributions is provided in the Methods. (**K**) Cumulative density of |*Δ*^*r*^*v*| for the simulation with RBCs and the *in vivo* measurements. Filling indicates regions where the cumulative density of divergent bifurcations is larger than that of convergent ones. For all *in vivo* measurements (D, F-G, K) the RBC velocity is displayed instead of the bulk flow velocity (superscript: RBC). Mouse icon: *in vivo* measurements. Computer icon: simulation results.

We measured RBC velocities in mice to analyse perfusion heterogeneity at capillary bifurcations (Fig 1A–1C, S1 and S2 Figs). The measurements were performed in all vessels of 70 randomly chosen bifurcations (Methods). Qualitative comparison of the velocities in the daughter vessels of divergent bifurcations reveals that at individual bifurcations the outflow velocities are of similar magnitude (Fig 1D). This is an interesting result because globally the velocity distribution in the capillary bed is highly heterogeneous (S3 Fig) [12, 14–16]. How can these similar outflow velocities be explained and what is different at convergent bifurcations?

To quantify the velocity difference at individual bifurcations we define the relative velocity difference

$$|\Delta^{r}v|=\frac{2|v^{b_{1}}-v^{b_{2}}|}{v^{b_{1}}+v^{b_{2}}}.$$ (1)

At divergent bifurcations $v^{b_{1}}$ and $v^{b_{2}}$ are the velocities in each daughter vessel while at convergent bifurcations the velocities in the mother vessels are used. The flow field and the instantaneous relative velocity difference fluctuate in time (Fig 1D) [14, 23, 46–50]. Please note, that the following studies are based on the time-averaged flow field (Methods), i.e. the relative velocity difference is computed from the median velocity in the individual capillaries.

Comparing the |*Δ*^*r*^*v*|-distribution for divergent and convergent bifurcations shows that the relative velocity difference at divergent bifurcations is significantly smaller than at convergent ones (Fig 1F–1G and 1K, Methods, p-value = 0.037, one-sided Mann-Whitney U Test).

We introduce the generic terms *well-balanced* and *unbalanced bifurcations* for bifurcations with a relative velocity difference < 20% and > 40%, respectively. These categories will be used to compare the flow dynamics at bifurcations with different relative velocity differences. The threshold value for *well-balanced bifurcations* is chosen based on the raw data for the *in vivo* measurements at divergent bifurcations (Fig 1F, lower plot). At levels above approximately 20% the distribution of the raw data is less dense. Additionally, the difference between the histograms for divergent and convergent bifurcations is largest for |*Δ*^*r*^*v*|<20% (Fig 1F–1I). The threshold for *unbalanced bifurcations* was chosen to clearly separate the two bifurcation categories from each other. It is important to note that the transition from *well-balanced* to *unbalanced bifurcations* is smooth and does not have a precise cut-off value. The threshold value of 20% has only been introduced for quantitative analysis.

To support our *in vivo* result we analyse the relative velocity difference at all divergent and convergent capillary bifurcations in our blood flow simulations with realistic MVNs. The numerical model has been introduced by Schmid et al. [12] and is summarized in the Methods. In brief, for a known distribution of RBCs we use an adjusted version of Poiseuille’s law [41] to compute the vessel resistance. Based on the continuity equation we set up a system of linear equations, which we solve for the pressure. The model is a close approximation to the situation *in vivo*, i.e. the presence of RBCs increases the flow resistance (Fahraeus-Lindqvist effect) and the RBCs distribute with a different ratio than the bulk flow (phase separation).

Indeed, our flow simulations in the two realistic MVNs (S4 Fig) from the mouse cortex confirm the reduced relative velocity difference at divergent bifurcations (Methods, Fig 1H–1I). A detailed comparison of the distributions of the relative velocity difference for the convergent and divergent bifurcations and for *in vivo* measurements and simulations is provided in the Methods.

Fig 1E shows an example, where the outflow velocities in the daughter vessels fluctuate around the perfectly balanced flow situation. The negative correlation between the two outflow velocities is a direct consequence of the alternating distribution of RBCs at the divergent bifurcation. This behaviour has already been observed at individual bifurcations in simulations in small MVNs [23, 33] and recent direct numerical simulations nicely demonstrate how the outflow velocities affect the RBC lingering at the bifurcation [23]. However, to the best of our knowledge, it has not yet been investigated to which extent the velocity balancing takes place on the network scale. Based on our simulations 26% of all divergent bifurcations are considered *well-balanced* (Fig 1H) and for 9% the relative velocity difference is even smaller than 5%.

It is important to note that at most bifurcations no negative correlation between the outflow velocities can be identified from the results (S6C and S6E Fig). That is, a velocity increase in one daughter vessel is usually not reflected by a simultaneous decrease in the other daughter vessel. We suspect that this is due to the combination of dynamic effects occurring simultaneously at other bifurcations in the vicinity. Further, investigations of the transient flow field are necessary to prove the existence of a negative correlation between the outflow velocities at divergent capillary bifurcations.

We conjecture that RBC dynamics play a key role in reducing the relative velocity difference at divergent bifurcations for the following reasons: First, because the fundamental difference between the two bifurcation types is that at divergent bifurcations RBCs are distributed while at convergent ones they are reassembled. Second, RBCs increase the flow resistance of capillaries [35] and thus the RBC distribution has a large impact on the flow field [23, 32, 33].

Please note, that all analysis presented in the following sections are based exclusively on the results of our blood flow simulations in realistic MVNs.

### The impact of RBCs on outflow velocities at divergent bifurcations

To confirm that RBC dynamics induce the velocity balancing we use two different numerical models to simulate blood flow in the realistic MVNs (Methods). As described above, the first model (with RBCs) is a close approximation of the situation *in vivo*. In the second numerical model RBCs are treated as passive particles (with pPs) that do not affect the vessel resistance and which, at divergent bifurcations, distribute with the same ratio as the bulk flow.

For the simulation with pPs the median relative velocity difference at divergent bifurcations is 74% and for the simulation with RBCs it is 56%. Consequently, we find that with pPs the median velocity difference is significantly larger than for the simulation with RBCs (Fig 1H and 1J, p-value = 5.1e^-58^, one-sided Mann-Whitney U Test). Moreover, there are fewer *well-balanced bifurcations* ($\left|\Delta^{r}v_{db}\leq 20\%\right|$) in the simulation with pPs (15%) than in the simulation with RBCs (26%, Fig 1H and 1J).

**Fig 2:**
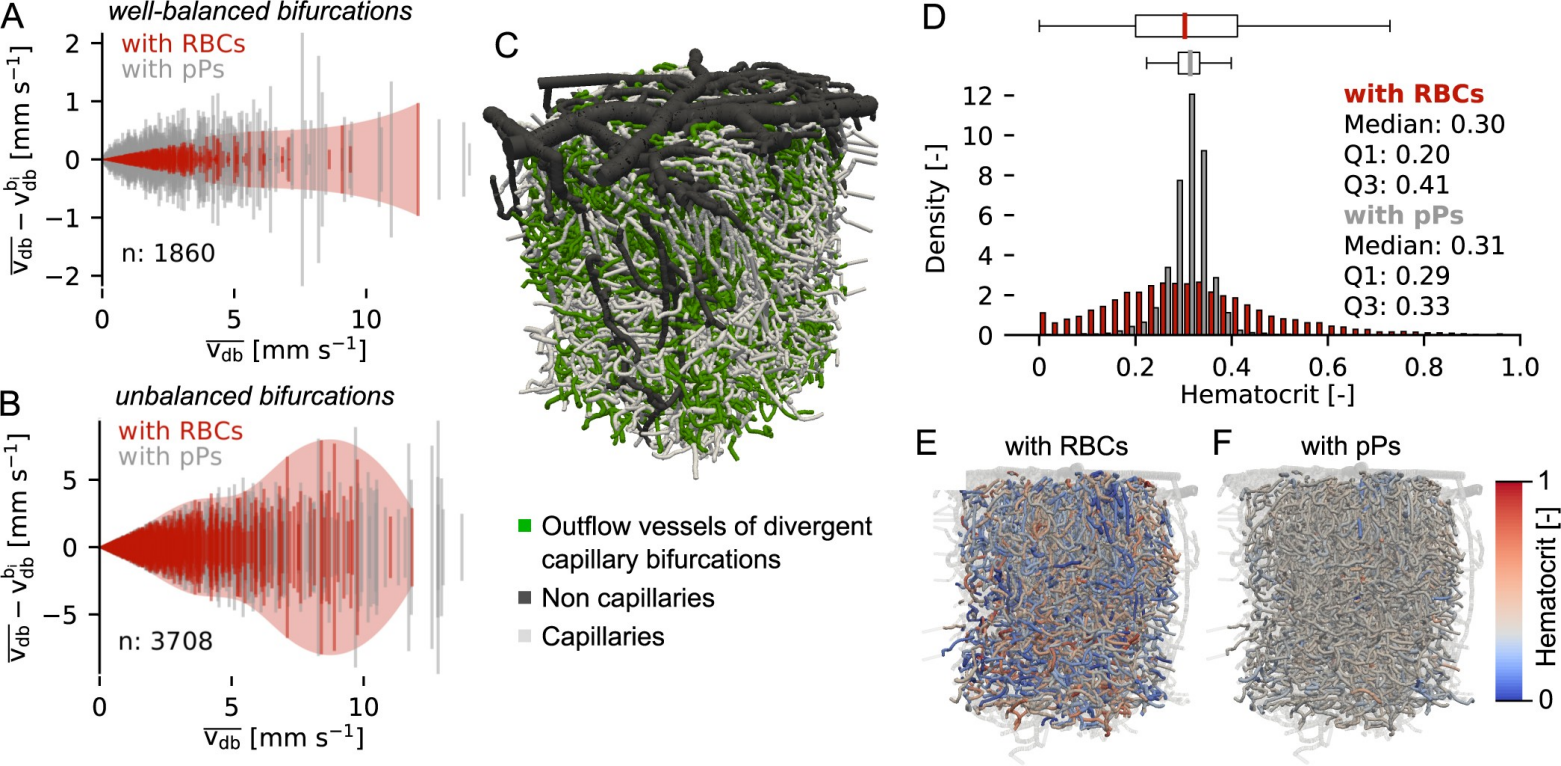
The impact of red blood cells balances outflow velocities at divergent capillary bifurcations and increases hematocrit heterogeneity in the capillary bed. (**A-B**) Absolute velocity difference at *well-balanced* and *unbalanced* divergent bifurcations for the simulation with red blood cells (RBCs) and with passive particles (pPs). $v_{db}^{b_{i}}$: bulk flow velocity in each daughter vessel of the bifurcation. $\bar{v_{db}}$: mean velocity in the daughter vessels. n: total number of bifurcations. The bifurcations are grouped into *well-balanced* and *unbalanced* based on the results from the simulation with RBCs. The same sets of bifurcations are compared for both simulations. We only depict velocities up to 14 mm/s (represents > 99.9% of all bifurcations). (**C**) Microvascular network 1 (MVN 1). The colouring highlights capillaries and outflow vessels of capillary divergent bifurcations. (**D**) Hematocrit distribution for the simulation with RBCs and with pPs (n = 13,988, Q1: lower quartile, Q3: upper quartile, Definition boxplot: Methods). (**E-F**) Hematocrit distribution in MVN 1 for the simulation with RBCs (E) and with pPs (F). In all plots only the daughter vessels of divergent bifurcation are considered (‘outflow vessels’, 53% of all capillaries, Methods, S8 Fig). In (A), (B) and (D) the data from MVN 1 and 2 is combined.

In addition to this, we analyse the absolute velocity difference at divergent bifurcations for the simulation with RBCs and with pPs (Fig 2A and 2B) at *well-balanced* and *unbalanced bifurcations* ($\left|\Delta^{r}v_{db}>40\%\right|$). We evaluate the set of bifurcations that are divergent in both simulation setups (91%). In total there are 7,285 divergent bifurcations in the simulation with RBCs.

Comparing Fig 2A and 2B reveals that the impact of RBCs on the balancing is larger at *well-balanced bifurcations* than at *unbalanced bifurcations*. This becomes apparent if we look at the bifurcations that are *well-balanced* in both numerical experiments. In this subset of bifurcations the relative velocity difference is reduced on average by 32% due to the presence of RBCs (S7 Fig, p-value = 6.2e^-22^, Wilcoxon signed-rank test). The corresponding analysis for *unbalanced bifurcations* yields a reduction in the relative velocity difference by only 8% (S7 Fig, p-value = 1.3e^-113^, Wilcoxon signed-rank test). A p-value of 1.8e^-18^ confirms that the velocity reduction at *well-balanced bifurcations* is significantly larger than at *unbalanced* bifurcations (Mann-Whitney U Test, S7 Fig).

Altogether, our results indicate that the presence of RBCs does not only lead to a larger number of *well-balanced bifurcations*, but that RBCs generally reduce the velocity difference at divergent bifurcations. These results confirm the balancing of outflow velocities at divergent bifurcations (Fig 1D–1I, Fig 2A and 2B) and clearly identify RBC dynamics as the main source of this effect (Fig 1H and 1J, S6 Fig).

### The impact of RBCs on hematocrit heterogeneity

Hematocrit heterogeneity is another relevant characteristic of the perfusion of the capillary bed, because it is directly related to the oxygen supply capacity of the microvasculature [17, 21, 26, 51]. As the precise mechanisms leading to this heterogeneity on the network scale are still poorly understood we aimed to separate heterogeneities resulting from topology from those attributable to the presence of RBCs. Once again, we used the simulation setup with RBCs and with pPs.

We analyse the hematocrit distribution in the outflow vessels of capillary divergent bifurcations (Fig 2C). While the hematocrit distribution is relatively flat for the simulation with RBCs, the distribution for pPs has a pronounced peak at the level of the inflow hematocrit and shows a smaller variance (Fig 2D–2F). *In vivo* measurements confirm a flat hematocrit distribution and thus agree qualitatively with the simulation with RBCs [17]. Consequently, the phase separation in combination with the Fahraeus-Lindqvist effect (Methods) is likely to be the key source of hematocrit heterogeneity (S8 Fig).

This observation agrees with the results of the direct numerical simulation of bi-phasic blood flow by Balogh and Bagchi [23], where it has been shown that hematocrit heterogeneity is a direct consequence of RBC lingering at bifurcations. It is important to note, that in contrast to Balogh and Bagchi [23] our model does not resolve RBC deformations but uses a simplified bifurcation rule to describe the motion of RBCs at divergent bifurcations (Methods). The qualitative agreement between the two works provides evidence that our simplified bifurcation rule captures the dominant aspects of phase separation. A more quantitative comparison would be necessary to further comment on the accuracy of our simplified description.

A prerequisite for the phase separation is an unequal flow partitioning at divergent bifurcations. This heterogeneous flow field is caused by the vascular topology and as such the vasculature indirectly contributes to the resulting hematocrit heterogeneity.

In summary, the impact of RBCs leads to globally increased hematocrit heterogeneity while it simultaneously locally reduces the velocity heterogeneity.

### The role of *well-balanced bifurcations*

So far, we have shown that the impact of RBCs balances the outflow velocities at divergent bifurcations. But what are the benefits of the velocity balancing at divergent bifurcations? In a previous numerical study, *well-balanced bifurcations* proved to be useful for a localized up-regulation of RBCs [32]. This suggests they might be relevant for regulatory purposes at the capillary level. Consequently, in the second part of this work we focus on identifying the role of *well-balanced bifurcations*. In line with this, we investigated the potential of small-scale regulation by capillary dilation.

To this end, we modified 25 *well-balanced* and 35 *unbalanced bifurcations* by dilating the diameter of one daughter capillary by 10% [9] (Methods, further dilation factors in S9 Fig). Each daughter vessel has been dilated in a separate simulation. Thus, in total we simulated 120 distinct capillary dilation scenarios and compared the relative change in flow rate and in number of RBCs. As the relative change in flow rate is also affected by the length of the dilated segment (S11 Fig), the relative change in flow rate is normalized with the length of the dilated segment and multiplied by 100 μm, i.e. we compare the relative change in flow rate with respect to an equivalent dilation along a 100 μm segment (Methods). To investigate the role of phase-separation during capillary dilation we introduce a further modification of our numerical model where we only turn off the phase-separation but keep the effect of RBCs on the vessel resistance (Methods).

**Fig 3:**
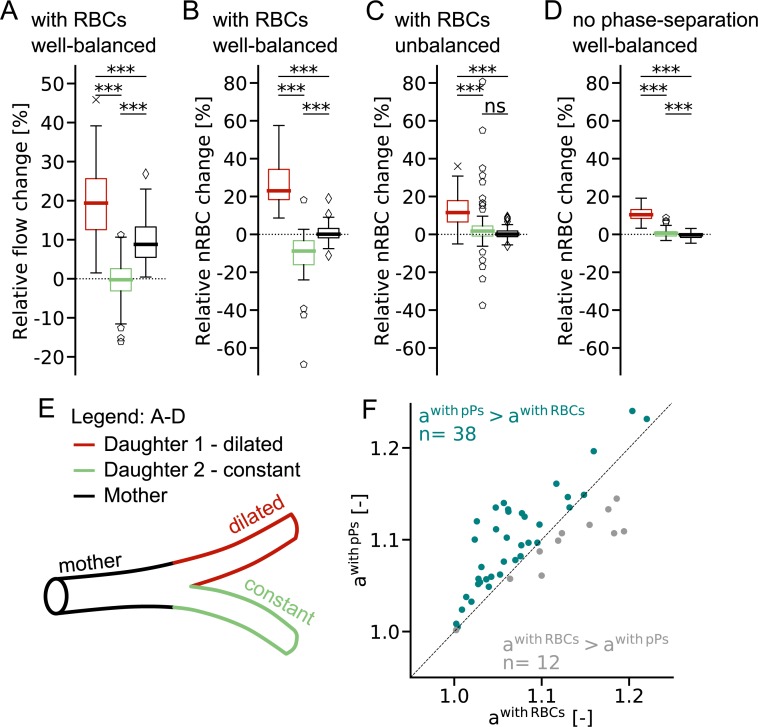
Capillary dilation locally increases the flow rate and the number of red blood cells. (**A-D**) Simulated relative change in flow rate (A) and in the number of red blood cells, nRBC (B-D) in response to a capillary dilation of 10% along a 100 μm capillary segment. (A-B): with red blood cells (RBCs), dilation at *well-balanced bifurcation*, (C): with RBCs, dilation at *unbalanced bifurcation*, (D): without phase separation, dilation at *well-balanced bifurcation*. Colour legend in (E). A sensitivity analysis on the threshold values for *well-balanced* and *unbalanced bifurcations* is provided in S12 and S13 Figs. (**E**) Schematic of divergent capillary bifurcation. (**F**) Quotient a^with pPs^ of flow ratios for the simulation, with passive particles (pPs) as a function of the quotient a^with RBCs^ of flow ratios for the simulation with RBCs for *well-balanced bifurcations*. Turquoise: a^with pPs^ > a^with RBCs^, grey: a^with RBCs^ > a^with pPs^. (A-B), (D), (F): Results from 50 capillary dilations. (C): Results from 70 capillary dilations. Statistical significance: Wilcoxon signed-rank test, ***: p<0.001, ns: non-significant (Methods). Definition boxplot: Methods.

For all scenarios the largest change in flow rate and in the number of RBCs occurs in the dilated vessel itself (Fig 3A–3D, S10 Fig). The largest average change in the number of RBCs in the dilated vessel is found at *well-balanced bifurcations* for the simulation with RBCs (27%, Fig 3B–3D, S2 Table). In the second daughter vessel the flow rate remains constant and the number of RBCs decreases (-11%, S1 Table). This specific redistribution of RBCs, as well as the preservation of flow in the second daughter vessel, is only present at *well-balanced bifurcations* (S10 Fig). Moreover, the significant increase in the number of RBCs in the dilated vessel can only be obtained if phase-separation is active (Fig 3B and 3D).

To study whether the chosen threshold for classifying *well-balanced* and *unbalanced bifurcations* affect the average values of the relative change in response to capillary dilation, we performed a sensitivity analysis on the impact of the threshold on the results presented in Fig 3 (S12 and S13 Figs). Changing the threshold by ± 10% does not lead to significant differences in the results. As previously stated, the bifurcation categories are mostly introduced for analysis purposes, because it facilitates the comparison of bifurcations with a small and a large relative velocity difference. Effectively, there is no precise cut-off value but a smooth transition from *well-balanced* to *unbalanced bifurcations*. This implies that the trends described will be more pronounced the more *well-balanced* or *unbalanced* the bifurcation is.

To study the impact of RBCs during capillary dilation, we analyse how the flow ratio at *well-balanced* divergent bifurcations changes in response to capillary dilation. The flow ratio is defined as the flow rate in the dilated vessel divided by that in the mother vessel (r = q^d1^/q^mother^). The quotient of flow ratios

$$a^{with\;RBCs}=\frac{r_{dilation}^{with\;RBCs}}{r_{baseline}^{with\;RBCs}}$$ (2)

compares the flow ratio during baseline and activation (e.g. capillary dilation) and is > 1 for all scenarios (Fig 3F), e.g. the fractional flow in the dilated vessel increases for all cases.

The impact of RBCs becomes most apparent if we compare the quotient of flow ratios for the simulation with RBCs and with pPs (Fig 3F). In 76% of the tested scenarios a^with pPs^ is greater than a^with RBCs^, that is, the flow ratio at *well-balanced* divergent bifurcations is changing less if RBCs are present.

In summary, the impact of RBCs leads to two beneficial effects during capillary dilation: (1) The changes in the RBC distribution are more pronounced at *well-balanced bifurcations*. Therewith, RBCs enhance the effectiveness of dilation (Fig 3B–3D). (2) The presence of RBCs helps to preserve the baseline flow ratio (Fig 3F). Consequently, RBCs are crucial for efficient local up-regulation and for a robust perfusion of the capillary bed.

### Quantifying changes induced by capillary dilation

**Fig 4:**
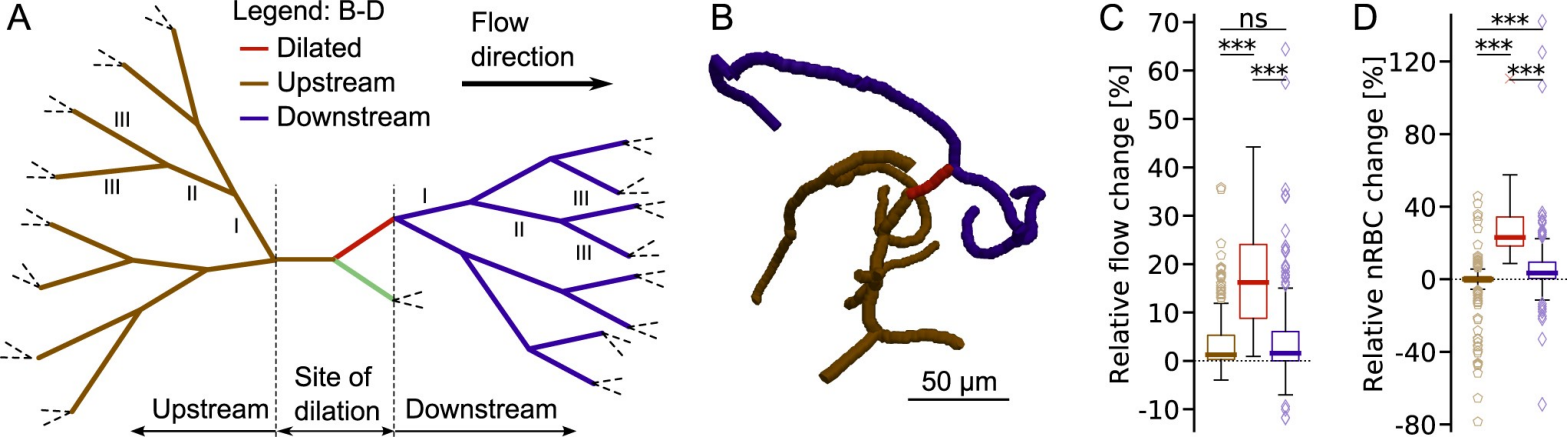
The simulated relative changes in response to capillary dilation are very local. (**A-B**) Vessels three generations up- and downstream of the site of dilation. (A) Schematic example. Roman numbers: generation up- and downstream of the site of dilation (one exemplary branch). (B) Realistic example from microvascular network 1 (MVN 1). (**C-D**) Simulated relative change in flow rate (C) and in the number of RBCs (nRBC) (D) for the dilated vessel and the vessels up to three generations up- and downstream of the site of dilation. Colour legend in (A). (C-D): Results from 50 capillary dilations at *well-balanced* divergent bifurcations. Statistical significance: Mann-Whitney U Test, ***: p<0.001, ns: non-significant (Methods). Definition boxplot: Methods.

To investigate the impact of capillary dilation on the surrounding network we examined the changes in all vessels three generations up- and downstream of the site of dilation (Fig 4A and 4B, Methods). The median relative change up- and downstream is < 5% for the number of RBCs and the flow rate (Fig 4C and 4D). Moreover, as for the previous analysis, the largest changes mostly occur in the dilated vessel itself. We conclude that the effects of single capillary dilation are very localized.

The relative change in flow rate depends not only on the dilation factor (S9 Fig) but also on the length of the dilated segment (S11 Fig) and on the overall network topology. For a capillary dilation of 10% we obtain an average increase in flow rate of 23% per 100 μm dilation. The average increase for 100 μm dilation is slightly smaller at *well-balanced bifurcations* (21%) than at *unbalanced bifurcations* (25%, S10 Fig). However, this difference is not statistically significant (p-value = 0.104, S2 Table). In the un-dilated second daughter vessel the flow rate does not change in response to capillary dilation at *well-balanced bifurcations*, while the flow rate decreases at unbalanced bifurcations (see S1 Table and S2 Table for statistics). This is another example of how the velocity balancing at divergent bifurcations and the presence of RBCs contribute to preserving the baseline flow rates.

The relative change in the number of RBCs is not a function of the length of the dilated segment (S11 Fig). The average increase for a 10% dilation is 27% at *well-balanced bifurcations* and 13% at *unbalanced bifurcations* (p-value = 3.15e^-10^, see Methods and S2 Table for details).

### Distribution of *well-balanced bifurcations*

If *well-balanced bifurcations* are crucial for robustness and for regulative purposes, they ought to be distributed throughout the cortical vasculature. Thus, our final investigations focus on the spatial distribution of *well-balanced bifurcations* in the MVN.

**Fig 5:**
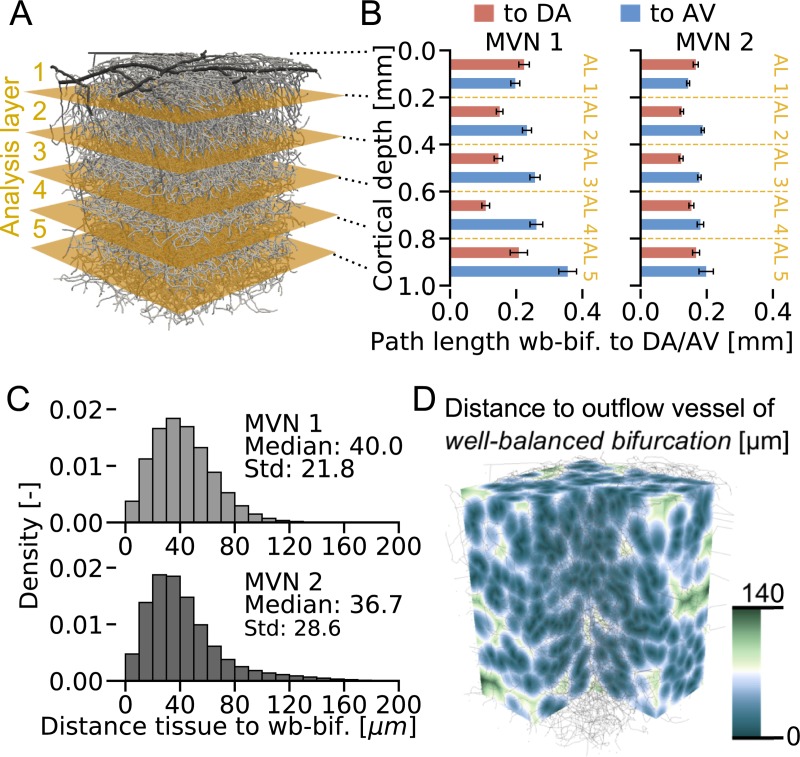
*Well-balanced bifurcations* are on average only 38 μm apart from any point in tissue. (**A**) Microvascular network 1 (MVN 1) and the five analysis layers (ALs) each 200 μm thick (Fig from [12]). (**B**) Minimum path length between *well-balanced bifurcation* (wb-bif.) and descending arterioles (DA)/ ascending venules (AV) for MVN 1 (left) and MVN 2 (right). The error bars show the standard error of the mean. The results of the statistical analysis are presented in S7 and S8 Tables. (**C**) Distribution of the distance from all tissue points to the closest outflow vessel of a *well-balanced bifurcation*. Upper plot: MVN 1, raw data in (D), Lower plot: MVN 2. (**D**) Distance for all tissue points to the closest outflow vessel of a *well-balanced bifurcation* in MVN 1. Distances >100 μm are mostly located at the border. The details to compute the variables shown in (B-D) are presented in the Methods.

First, we study differences with respect to cortical depth and divide the MVNs into five analysis layers (ALs), each 200 μm thick [12](Fig 5A). We computed: 1. the relative number of *well-balanced bifurcations* per AL, 2. the minimum Euclidean distance between *well-balanced bifurcations*, 3. the minimum Euclidean distance between *well-balanced bifurcations* and descending arteriole (DA)/ascending venule (AV) and 4. the minimum path length from *well-balanced bifurcation* to DA and AV (Fig 5B, S14 Fig, Methods). It is important to note that these characteristics can also be affected by topological differences over depth.

The relative number of *well-balanced bifurcations* per AL varies between 26% and 40% for MVN 1 and between 33% and 39% for MVN 2 (S14A Fig). While in MVN 1 we observe a minimum in the relative number of *well-balanced bifurcations* for AL 3, this trend is not confirmed in MVN 2. As the coefficient of variation is 15% for MVN 1 and 5% for MVN 2, we conclude that the relative number of *well-balanced bifurcations* does not vary significantly over depth. The Euclidean distance between *well-balanced bifurcations* decreases on average by 16% from AL 1 to AL 2 (S14B Fig, S4 Table) and remains approximately constant in the layers below.

The results for the Euclidean distance and the minimum path length between *well-balanced bifurcations* and the closest penetrating vessel exhibit similar trends. For the Euclidean distance and the minimum path length between *well-balanced bifurcations* and DA (S14C Fig, Fig 5B, S5 Table, S7 Table) we observe the shortest distances/ path lengths for AL 2–4. The median Euclidean distance of AL 1 and AL 5 is on average 28% larger than the median distance of AL 2–4. For the minimum path length we find an average difference of 43% between AL 2–4 and AL 1 and AL 5.

For the Euclidean distance between *well-balanced bifurcations* and AV we observe an increase over depth (S14D Fig, S6 Table). In MVN 1 this trend is similar for the minimum path length between *well-balanced bifurcations* and AV. However, in MVN 2 the minimum path length increases from AL 1 to AL 2 but no significant changes are notable for the layers below (Fig 5B, S8 Table).

Taken together, most characteristics show a homogeneous distribution between AL 2–4 and the largest differences are observed with respect to AL 1 and AL 5. For the distance and the minimum path length between *well-balanced bifurcations* and AV we observed an increase over depth.

Comparing the minimum path lengths from *well-balanced bifurcations* to DA and to AV shows that in AL 2–5 *well-balanced bifurcations* are on average 91 μm closer to the DA (Fig 5B, S14E Fig, S9 Table). This configuration is plausible for two reasons: 1. Preserving robust perfusion is more relevant at locations further upstream and 2. Contractile mural cells are only present close to DAs [52, 53].

Fig 5C shows the distributions for the distance to the closest outflow vessel of a *well-balanced bifurcation* in MVN 1 and 2 (Fig 5D), respectively. A median distance of 40.0 μm (MVN 1) and 36.7 μm (MVN 2) proves that most tissue points are close to a *well-balanced bifurcation*. If we define a region of influence based on the average inter-capillary distance (~50 μm) [44], 69% of the tissue can be effectively influenced by capillary dilation.

## Discussion

We identified the balancing of outflow velocities at divergent bifurcations as key element of microvascular flow, which is directly induced by RBC dynamics. This result is important for various aspects related to microvascular perfusion and is likely an evolutionary benefit of the bi-phasic nature of blood. First, our study provides evidence for the significant role of RBC dynamics and thereby confirms existing theoretical considerations [32, 40, 43]. Second, from a functional point of view, *well-balanced bifurcations* are relevant for regulatory purposes. This hypothesis is reinforced by the convenient spatial distribution of *well-balanced bifurcations* and the more efficient up-regulation of RBCs during capillary dilation.

We hypothesize that different bifurcation types (*well-balanced* and *unbalanced*, convergent and divergent) fulfil different regulatory tasks. It seems likely that those differences are also reflected anatomically, for example in mural cell types, mural cell densities or different diameter distributions at the bifurcation. A recent study shows that pericyte morphology differs significantly along the capillary path [53] and consequently different functional tasks seem plausible. Further studies are necessary to refine the role and the development of *well-balanced bifurcations*.

The velocity balancing at individual bifurcations indicates that RBCs dampen the flow field. Based on the physics of the RBC dynamics, RBCs distribute to minimize the outflow velocity differences at bifurcations. However, this mechanism is very local, and decreasing the velocity difference at one bifurcation might increase it at another bifurcation. Additionally, the number of available RBCs and a large difference in flow rates can limit the balancing. As a result, the flow field and the RBC distribution seem to fluctuate around the most *well-balanced* state possible.

As stated previously, we focused on the time-averaged flow field. Nonetheless, we want to point out that the analysis of the transient flow field could allow further insights on the flow dynamics at capillary bifurcations. In our numerical model the velocity fluctuations are a direct consequence of the fluctuating RBC distribution. Consequently, the analysis of transient changes is an important aspect for a profound understanding of the impact of RBCs on the flow field. Especially if we keep in mind, that temporal fluctuations are most pronounced in the capillary bed [23]. Taken together, our knowledge on temporal fluctuations in microvascular flow remains limited. We believe that a profound analysis of the transient flow field in large realistic MVNs needs to be performed to provide the basis for future investigations.

By analysing the preservation of the flow ratio in response to capillary dilation we extended our studies addressing the damping effects of RBCs. We observed that the impact of RBCs reduces the change in flow ratio at *well-balanced bifurcations*. It is important to note that this result is not only valid for capillary dilations, but also for any downstream alteration that induces a change in flow rate. However, preserving the flow ratio comes at the cost of local alterations in RBC distribution. This implies that under certain circumstances preserving perfusion might be more crucial than preserving the RBC distribution.

Altogether we suggest that both mechanisms (RBC up-regulation and preservation of the flow ratio) are important for well-adjusted oxygen and nutrient supply. The most fascinating aspect of the described mechanisms is that the impact of RBCs is an intrinsic feature of the bi-phasic nature of blood.

Our results confirm the global hematocrit and RBC velocity heterogeneity in the capillary bed [14–22]. While the global RBC velocity heterogeneity is caused by the vascular topology, the global hematocrit heterogeneity results from the heterogeneous flow rates in combination with the RBC dynamics. Interestingly, the local velocity balancing by RBCs is not visible at the network scale.

What are the possible benefits of the heterogeneous perfusion for nutrient supply? The existing hematocrit heterogeneity bears potential for an increase in oxygen extraction fraction by hematocrit homogenization [24]. However, this advantage might come at the risk that areas with low hematocrit values are more sensitive to tissue hypoxia. Furthermore, hematocrit heterogeneity will increase the heterogeneity in oxygen saturation of RBCs. As such, it affects the tissue cylinder radius supplied by a capillary and the diffusive interaction between RBCs [54, 55].

The RBC velocity heterogeneity might be necessary for the redistribution of flow during activation. This hypothesis is fostered by *in vivo* measurement that show that high and low flux capillaries respond differently to neuronal activation [19, 22, 29, 47] and that RBC velocity increases as well as decreases can be observed [16]. This redistribution of flow leads to a reduction of capillary transit time heterogeneity (CTH) [18, 19, 29, 47]. We suggest that flow homogenization is a secondary regulation mechanism that together with the overall up-regulation of flow refines nutrient supply during activation.

Our functional study on the impact of capillary dilation reveals that single capillary dilation is able to locally alter perfusion and RBC distribution. As previously mentioned, it is essential that the level of the relative change does not only depend on the dilation factor and the dilated vessel length, but also on the entire vascular topology. This can be explained by fact that the cortical vasculature is comparable to an interconnected resistor network, where each local change affects the entire network. Nonetheless, we are able to show that the largest changes occur in the dilated vessel and as such the effects of capillary dilation can be considered to be quite local.

We also want to underline that oxygen availability is governed by the RBC velocity and the hematocrit (e.g. number of RBCs). Only few *in vivo* studies measure both quantities [17, 21, 37]. Frequently only RBC velocity changes in response to stimulation are considered. However, the RBC velocity alone gives only limited insight on the change in RBC flux. In the olfactory bulb glomeruli it has been shown that the RBC velocity increases in response to stimuli [37]. However, for the hematocrit increases as well as decreases have been observed [37]. Our results show that capillary dilation has a large effect on the RBC distribution. This may suggest that capillary dilation might be more relevant for altering the RBC distribution than for increasing the flow rate. Therefore, we hope that changes in RBC distribution will be addressed more frequently in *in vivo* measurements.

To facilitate comparison, we only analysed the impact of single capillary dilations. However, it has been observed *in vivo* that multiple capillaries respond simultaneously. Additionally, the response pattern can vary between capillaries and even along capillaries [56]. While the impact of such a response scenario is more difficult to study, it also bears the potential for larger and more sophisticated adaptations of flow and RBC distributions.

For the presented results it is irrelevant, whether a capillary dilates actively or passively, or whether the vessel resistance is reduced by an increased RBC deformability [57]. For active and passive capillary dilation pericytes are the mural cells of interest. A reduced pericyte tone could directly induce capillary dilation or lead to a larger vessel distensibility, which subsequently would result in vasodilation.

In summary, we suggest that the bi-phasic nature of blood is a convenient intrinsic feature that increases robustness of perfusion and facilitates regulation. Capillary dilation proves to be an efficient mechanism to locally alter perfusion and RBC distribution. Consequently, from a functional point of view, it seems likely that capillary diameters changes are relevant for neurovascular coupling.

## Materials and methods

### Ethics statement

Surgical and experimental procedures, as well as animal husbandry protocols, were approved by the Veterinary Office, Canton of Zürich, and performed according to Swiss law (Federal Act on Animal Protection 2005 and Animal Protection Ordinance 2008). For *in vivo* two-photon imaging the mice were anaesthetized using the triple anaesthetic mixture (fentanyl 0.05 mg/kg, Sintenyl, Sintetica; midazolam 5 mg/kg, Dormicum, Roche; and medetomidine 0.5 mg/kg, Domitor, Orion Pharma), re-administered after ~45 minutes for maintenance of anaesthesia.

### Animals and surgical preparation

We used six adult, female C57BL/6J mice (Charles River) for *in vivo* imaging experiments. The mice were housed under an inverted 12-hour light/dark cycle, with food and water *ad libitum*, in cages of 2–4 littermates. At the time of the first surgical procedure, the mice were 8–12 weeks old, weighing 20-25g.

The surgical procedures used to prepare the mice for imaging have been described previously [58]. Under isoflurane anesthesia (AbbVie, 4% for induction, 1–2% for maintenance), a custom-made aluminium headpost was attached to the skull using dental cement (Synergy D6 Flow, Coltene, cured using blue light). After 24–48 hours, using a triple anaesthetic mixture (fentanyl 0.05 mg/kg, Sintenyl, Sintetica; midazolam 5 mg/kg, Dormicum, Roche; and medetomidine 0.5 mg/kg, Domitor, Orion Pharma), a craniotomy was performed over the somatosensory cortex and a 3x3mm sapphire glass coverslip (Valley Design) was positioned over the exposed brain and secured with more dental cement. The mice were given pain relief (buprenorphine, 0.1mg/kg s.c. every six hours during the day and in drinking water overnight, 0.3 mg/ml) for 3 days following surgery and were allowed to recover in their home cage for at least one week before imaging.

### *In vivo* two-photon imaging

Surgical and experimental procedures, as well as animal husbandry protocols, were approved by the Veterinary Office, Canton of Zürich, and performed according to Swiss law (Federal Act on Animal Protection 2005 and Animal Protection Ordinance 2008).

*In vivo* imaging experiments were performed with a custom-built two-photon microscope [59]. The mice were anaesthetized using the triple anaesthetic mixture described earlier, with midazolam (5 mg/kg) re-administered after ~45 minutes for maintenance of anaesthesia. The blood plasma was labelled with FITC-Dextran (5%, 59-77kDa, Sigma) injected via the tail vein. In plane bifurcations up to ~300 μm below the cortical surface were randomly selected for imaging. A total of 38 diverging and 32 converging bifurcations were used in the study. Ideally, the RBC velocity would be measured simultaneously in each vessel of the bifurcation. However, as this is technically not possible, each vessel was measured for three ~10 s windows, interleaved with measurements of the other vessels from the same bifurcation. This measurement protocol ensured that our individual measurements are steady over a time window of 60 s. Therewith, we are confident that our individual measurements are representative for the time-averaged flow field at the bifurcation. The consecutive line-scans were performed immediately after one and another.

### Analysis procedure of the line-scans

The RBC velocities have been calculated with the Radon-transform-algorithm [60] implemented in the open-source MATLAB toolbox CHIPS [61] (Cellular and Hemodynamic Image Processing Suite). For each measurement we use the median velocity as the representative velocity for this capillary. Generally, the agreement between the three consecutive measurements is very good (median difference is 5.5%, S1 and S2 Figs). However, as we tried to keep averaging to a minimum, we use the RBC velocity obtained in the second *in vivo* measurement.

### Microvascular networks

The numerical simulations are performed in two realistic microvascular networks (MVN) from the mouse parietal cortex [6]. Generally, each MVN can be represented as a graph consisting of a set of nodes (bifurcations) connected by edges (vessels).

The realistic MVNs used in this study perfuse an approximately cubic domain representing a tissue volume of ~1 mm^3^. They were acquired with two-photon laser scanning microscopy [62, 63]. The labelling of vessels and the diameter distribution of the original MVNs have been slightly modified [12]. The vessels are labelled to differentiate between pial arterioles, descending arterioles, capillaries, ascending venules and pial venules. In order to assign the vessel types, the topology and the vessel diameters are taken into account. Further information on the MVNs under investigation can be found in Blinder et al. [6] and Schmid et al. [12].

### The numerical model and its variations

The numerical model was first described in Obrist et al. [43] and Schmid et al. [32] and was extended to large realistic MVNs in Schmid et al. [12]. For an in depth description please consult Schmid et al. [12, 32].

The flow rate in each vessel is computed by Poiseuille’s-law

$$q_{ij}=\frac{p_{i}-p_{j}}{R_{ij}^{e}},$$ (3)

where q_ij_ is the flow rate in the vessel connecting nodes i and j; p_i_ and p_j_ are the respective pressure values at these nodes. The effective resistance $R_{ij}^{e}$ is a function of the hematocrit [35]. For pure plasma flow in a vessel with circular cross section the resistance is

$$R_{ij}=\frac{128\mu L_{ij}}{\pi D_{ij}^{4}},$$ (4)

where L_ij_ and D_ij_ are the length and the diameter of vessel ij and μ is the dynamic viscosity of blood plasma. The mass balance at every node i in combination with boundary conditions for each in- and outflow result in a system of linear equations that can be solved for the pressure.

The distribution of RBCs influences the flow and pressure fields and vice versa. Three RBC related phenomena must be considered to accurately model the bi-phasic character of blood: 1. the Fahraeus-Lindqvist effect [35, 41], 2. the Fahraeus effect [41, 64] and 3. the phase separation at vessel bifurcations [12, 65].

The Fahraeus-Lindqvist effect describes the impact of RBCs on the resistance of the vessel [35]. We use the empirical formulation derived by Pries et al. [41] to account for RBCs. The second RBC-related flow phenomenon is the Fahraeus effect [64], which leads to a reduced tube hematocrit because RBCs move on average faster than the bulk flow as they tend to travel in the centre of the vessel [66, 67]. In our simulations we use an empirical function by Pries et al. [41] to account for the Fahraeus effect.

Phase separation is a phenomenon that is mostly relevant at divergent capillary bifurcations [65]. It states that the fractional plasma- and RBC-flows are not equal in the daughter vessels, i.e. RBCs are distributed with a different ratio than plasma. This effect is most pronounced if the diameter of the mother vessel is < 10 μm. Consequently, in our numerical model we use two distinct formulations to describe the phase separation. For vessels 10 ≥ μm we use the empirical equations by Pries et al. [65]. In vessels < 10 μm we assume that RBCs follow the path of the largest pressure force (bifurcation rule) [12, 32]. This assumption is based on a simplified analysis of the forces on a single RBC at divergent bifurcations and is justified in more detail in Fung [40] or Schmid et al. [12].

Along with the bifurcation rule, the motion of RBCs at divergent bifurcations can be affected by RBC ‘traffic jams’. At convergent bifurcations traffic jams occur if two RBCs arrive at approximately the same time. At divergent bifurcations the different RBC velocities in the daughter vessels (Fahraeus effect) can lead to RBC jams in the mother vessel or a distribution of RBCs that does not follow the bifurcation rule.

For simulations with RBCs, all RBC-related flow phenomena are considered. To test the impact of the different phenomena we work with variations of the standard simulation setup. In the simulations with passive particles (with pPs) we switch off the Fahraeus-Lindqvist effect and the phase separation. Thus, RBCs do not influence the resistance of the vessel and at divergent bifurcations they distribute with the same ratio as the bulk flow except for traffic jam effects. In the simulations without phase separation we only switch off the phase separation but keep the impact of RBCs on the vessel resistance. These different simulation setups allow us to separate effects resulting from the phase separation and from the Fahraeus-Lindqvist effect.

It is important to note, that some uncertainty exists with respect to the empirical equations used to model the hydrodynamical effects of RBCs. However, as long as the general trends described are correct our qualitative results will not be affected. For example, if the impact of RBCs on the vessel resistance would be larger than predicted by the empirical equations currently used we would still observe a velocity balancing at divergent bifurcations. In this case, the velocity balancing would most likely be even more pronounced than in our current setup.

Recent evidence suggests that especially in smaller vessels a deviation from Poiseuille’s law is possible [23]. This deviation occurs temporarily in situations where a lingering RBC temporarily blocks downstream vessels. In small vessels the blockage can be very frequent and consequently also the time-averaged relation between pressure drop and flow rate can be affected. We do not account for the deviation from Poiseuille’s law in our numerical model. However, vessel blockage is a temporary effect and for most vessels the correlation between pressure drop and flow rate is positive [23]. Therefore, we are confident that neglecting the temporary deviation from Poiseuille’s law does not significantly affect our time-averaged analysis of the flow field.

### Boundary conditions

In order to solve the linear system of equations for the pressure we need boundary conditions at all in- and outflow vertices. In previous work [12] we developed and validated a new approach to assign suitable pressure boundary conditions. The inflow hematocrit is set to 0.3 at all inflow nodes [17, 68].

### Discrete RBC tracking

A special feature of our modelling approach is that we track RBCs individually. This allows us to describe their motion at divergent bifurcations based on simplified physical assumptions and reduces the amount of modelling by empirical relations. Moreover, RBCs have a finite volume and thus situations such as RBC traffic jams are modelled more accurately than if using infinitely small particles.

### Initial conditions and time-averaged flow field

The simulations are initialized with homogeneous hematocrit distributions. The simulation time step is set to 0.75 ms for MVN 1 and to 0.5 ms for MVN 2 (see Schmid et al. [12] for details). Over time, the hematocrit distribution and the flow field converge to their statistical steady state. Nonetheless, the hematocrit distribution and the flow field continue to fluctuate around the statistical steady state. For our subsequent analysis we use the time-averaged flow field and RBC distribution.

In order to define the averaging interval for our simulations we compute the turn over time for each vessel. The turn over time for a vessel is defined as the time necessary to completely perfuse the vessel once, e.g. the vessel length divided by the flow speed. Because of the large range of flow velocities the vessel turn over time of different vessels varies significantly.

Our averaging interval is chosen such that 90% of all vessels are completely perfused at least 10 times (averaging interval: MVN 1: 12 s, MVN 2: 5s). To ensure that the statistical steady state is reached, the simulations are run for at least two averaging intervals before we start the time averaging.

As the flow field in the simulations fluctuates strongly, we employ a moving average for the illustration of the time courses in Fig 1E and S6 Fig. The moving average is computed over 100 time steps and at every 50 time steps.

### Focus on outflow vessels of divergent capillary bifurcations

In our analysis we focus on outflow vessels of divergent capillary bifurcations. Two reasons motivate this approach. First, divergent bifurcations play a crucial role in the distribution of RBCs and blood flow. Second, by limiting our comparison to outflow vessels of divergent bifurcations we know that we are looking at comparable vessel types in experiments and simulations. It is important to note that in a few cases the bifurcation type (divergent or convergent) differs in the simulation with RBCs and with pPs.

### Comparison of the relative velocity differences

In Fig 1F–1I we compare the relative velocity difference at divergent and convergent bifurcations for the simulation with RBCs and for *in vivo* experiments. We postulate that the distributions are different for divergent and convergent bifurcations. To be more precise, we expect a lower median and a larger positive skew at divergent bifurcations. Both aspects are confirmed *in vivo* and in the simulation with RBCs (S3 Table). The larger skew is also reflected in the smaller value for the first quartile (Q1) for divergent bifurcations (S3 Table).

We use the one-sided Mann-Whitney U Test to test whether the median of the relative velocity difference at divergent bifurcations is smaller than at convergent bifurcations. Indeed, a p-value of 0.037 for the *in vivo* measurements confirms our hypothesis (simulation with RBCs: p-value = 3.2e^-70^). Additionally, we use the one-sided Kolmogorov-Smirnov-Test to compare the cumulative densities of the relative velocity difference between divergent and convergent bifurcations (Fig 1K). Here, a p-value of 0.042 reinforces that the cumulative density of the relative velocity difference is larger for divergent bifurcations, e.g. the distribution is more positively skewed (simulation with RBCs: p-value = 4.02e^-61^).

The general trends are in accordance for the *in vivo* measurements and the simulation with RBCs. However, the median and the standard deviation of the relative velocity difference are larger in the simulation with RBCs than in the *in vivo* experiments for both bifurcation types. Various aspects could cause these discrepancies. Regarding the *in vivo* measurements it is important to note that our sample size is relatively small and that we do not have any relative velocity differences greater than 130%. Additional *in vivo* measurements would be necessary to quantitatively comment on those differences. The absolute values of our numerical simulations could also be affected by various modelling assumptions. The most important ones are: (1) The chosen inflow hematocrit of 0.3 is on the low side. A higher inflow hematocrit leads to more RBCs in the MVN and thus increases the capability to balance velocity differences [32]. (2) The uncertainty in the vessel diameter estimates can have a significant impact on the local flow velocities at divergent bifurcations. (3) The empirical equations also affect the velocity balancing at divergent bifurcations.

Because of the existing uncertainties it is difficult to quantitatively compare *in vivo* measurements and numerical simulations with each other. Nevertheless, these uncertainties do not affect the comparison between divergent and convergent bifurcations within the same setup.

### Definition of boxplots

For all boxplots (Fig 2D, Fig 3A–3D, Fig 4C and 4D, S7–S10 Figs, S12 and S13 Figs) we use the following definitions: The box extends from the lower (Q1) to the upper quartile (Q3) of the underlying data. The thick line depicts the median. The upper and the lower whisker extend to the last data point < Q3 + 1.5(Q3-Q1) and to the first data point > Q1–1.5(Q3-Q1), respectively. Data points outside the range of the whiskers are illustrated as separate outliers.

### Performing capillary dilations

In order to perform representative capillary dilations, we define a set of criteria to choose suitable divergent bifurcations. All criteria are based on the time-averaged simulation results of the simulation with RBCs.

The first criterion is based on the relative velocity difference during baseline. Here, we either choose *well-balanced* ($|\Delta^{r}v_{db}|\leq 20\%$) or *unbalanced divergent bifurcations* ($|\Delta^{r}v_{db}|>40\%$). To avoid any impact from the boundary we ensure that the divergent bifurcation is relatively close to the centre of the MVN (i.e. the distance of the bifurcation to the centre of the MVN has to be smaller than 0.6 times the maximum distance of all nodes to the centre). As we are also interested in the role of RBCs during capillary dilation we select bifurcations where the hematocrit in the mother vessel is ≥0.3 such that sufficient RBCs are present.

From the set of suitable divergent bifurcations, we randomly choose 25 *well-balanced* and 35 *unbalanced* divergent bifurcations and check that they are well distributed over the whole depth of the cortex. For each of those bifurcations we perform two simulations, in which each daughter vessel has been dilated once. Thus, in total we simulated 50 distinct capillary dilation scenarios for *well-balanced bifurcations* and 70 scenarios for *unbalanced bifurcations*.

As previously stated, at divergent capillary bifurcations the RBCs follow the path of the largest pressure force. The pressure force is a function of the cross-section of the vessel and consequently, changing the capillary diameter at the bifurcation would significantly affect the bifurcation rule. To eliminate this effect, we keep the capillary diameter at the divergent bifurcation constant and only dilate a segment of the chosen capillary (S9 Fig). Therefore, the capillary is split into two segments. The length of the segment adjacent to the divergent bifurcation is set to six times the length of an RBC in that vessel.

The capillaries are dilated by 10%, i.e. the dilation factor is f_dil_ = D^dilated^/D^baseline^ = 1.1 [9], where D^baseline^ and D^dilated^ are the vessel diameters before and after dilation. Simulation results for further dilation factors are provided in S9 Fig.

It should be noted, that these results are not limited to capillary dilation but that they are also valid for other scenarios, where the resistance of individual vessels decreases, e.g. by more deformable RBCs [57].

The effects of capillary dilation are only studied in MVN 1.

### Capillary dilation results–Computation of relative changes

As previously stated, for most of our numerical analyses we use the time-averaged flow field and RBC distribution. To comment on the changes in response to capillary dilation (activation) we compute the relative difference for each vessel. As the change in flow rate is a function of the length of the dilated segment, the relative change

$$\Delta_{capillary\;Dil.}^{r}q_{ij}=\frac{q_{ij}^{activation}-q_{ij}^{baseline}}{q_{ij}^{baseline}}\cdot \frac{100\;\mu m}{L_{ij}^{dilated}},$$ (5)

is normalized by $100\;\mu m/L_{ij}^{dilated}$, where $q_{ij}^{baseline}$ and $q_{ij}^{activation}$ are the flow rates in vessel ij during baseline and activation, respectively. $L_{ij}^{dilated}$ is the difference between the original vessel length L_ij_ and the constant vessel length $L_{ij}^{constant}$ at the bifurcation (S9 Fig).

The relative change

$$\Delta_{capillary\;Dil.}^{r}{nRBC}_{ij}=\frac{{nRBC}_{ij}^{activation}-{nRBC}_{ij}^{baseline}}{{nRBC}_{ij}^{baseline}}$$ (6)

in the number of RBCs is not a function of the dilated segment length and thus normalization is not necessary.

To investigate how local the effects of capillary dilation are, we analyse all changes in the vessels three generations up- and downstream of the site of dilation. The site of dilation is defined as the whole bifurcation at which capillary dilation is performed, e.g. the mother and the two daughter vessels. All vessels that deliver blood to the mother vessel of the divergent bifurcation are upstream vessels of generation I. The bifurcations that bring blood to the vessels of generation I are upstream vessels of generation II and so on. The equivalent definition is used to define the vessels downstream of the site of dilation. Here, the first vessels downstream of the dilated capillary are downstream vessels of generation I (Fig 4A). Depending on the topology and the flow situation there can be from 3 to 39 vessels up-/downstream of the site of dilation. All up-/downstream vessels of the 50 capillary dilation scenarios are grouped together for the boxplots illustrated in Fig 4C and 4D.

### Capillary dilation results—Statistical analysis

The first step in analysing the relative changes in response to capillary dilation is to test which changes are in fact significantly different from 0. The resulting p-values are summarized in S1 Table.

If we want to compare the changes between different vessel types for one simulation setup, we need a statistical test that is suitable for paired samples (Fig 3A–3D, S9 and S10 Figs). Here, we chose a nonparametric test to compare the median values of the samples (Wilcoxon signed-rank test).

Next, we compare the relative changes between different simulation setups. As we want to understand for which scenario we observe the larger/smaller change, we use the one-sided Mann-Whitney U Test that allows for the provision of an alternative hypothesis (S2 Table).

To analyse the impact of capillary dilation on the proximity of the site of dilation we look at the changes in the vessels three generations up-/downstream of the site of dilation. As the vessels are not directly connected to each other the samples can be considered independent. Consequently, we use the two-sided Mann-Whitney U Test.

### Methods to analyse the distribution of *well-balanced bifurcations*

In order to analyse the distribution of *well-balanced bifurcations* we calculated different measures to describe their position in the cortical vasculature. Here, we provide additional information on how these measures have been computed.

Several of the subsequently described measures are given with respect to descending arterioles (DA)/ ascending venules (AV) or more precisely with respect to the main branch of DAs and AVs.

The first measure is the relative number of *well-balanced bifurcations*, which is the ratio of the number of *well-balanced divergent bifurcations* to the total number of divergent bifurcations. The minimum Euclidean distance between *well-balanced bifurcations*, the second measure, is computed by identifying the closest neighbouring *well-balanced bifurcation* for each *well-balanced bifurcation*. The third measure is the Euclidean distance between a *well-balanced bifurcation* and the closest point along a DA/AV.

Lastly in the forth measure we computed the minimum path length from *well-balanced bifurcation* to DA/AV. To compute the minimum path length, we move up-/downstream from each *well-balanced bifurcation* until the DA/AV is reached. For each *well-balanced bifurcation* we obtain a set of possible paths to the DA/AV. The shortest path length of this set is the minimum path length to the DA/AV for this *well-balanced bifurcation*. Based on the cortical depth of the *well-balanced bifurcation* the minimum path length is averaged for each of the five ALs.

Our final analysis regarding the distribution of *well-balanced bifurcations* is on the distance from each tissue point to the closest outflow vessel of a *well-balanced bifurcation*. Here, we need a discrete representation of the tissue in which the MVN is embedded. Therefore, the tissue is divided into 100x100x132 (MVN 1)/ 100x100x114 (MVN 2) cubes, which results in a cube size of 8.2x8.2x8.2 μm for MVN1 and 8.6x11.2x9.9 for MVN 2. Now, for each centre of the cube we calculate the distance to the closest outflow vessel of a *well-balanced bifurcation*. This results in 1,320,000/ 1,140,000 data points for the histograms in Fig 5C for MVN 1 and MVN 2, respectively.

## Supporting information

S1 Fig Raw data of red blood cell velocity measurement at divergent and convergent bifurcations *in vivo*. (**A**) Red blood cell (RBC) velocity in the daughter vessels of 38 divergent bifurcations (daughter vessel 1: x, daughter vessel 2: +). (**B**) RBC velocity in the mother vessels of 32 convergent bifurcations (mother vessel 1: x, mother vessel 2: +). (**C-D**) The relative difference to the mean velocity at the bifurcation is depicted for divergent (C) and convergent (D) bifurcations (Rel. dif. to mean velocity). The mean velocity of a bifurcation is computed as the average over the six measurements in the two outflow/ inflow vessels, respectively. In all subplots, for each value along the x-axis the measurements for one bifurcation are grouped. The RBC velocity for the three measurements per vessel is depicted. As stated in the Methods the agreement between the three consecutive measurement is good (median relative difference 5.5%). The colour map shows the relative RBC velocity difference for each bifurcation (Rel. RBC velocity difference). The definition of the relative velocity difference is provided in the main text. For illustrative purposes the bifurcations are ordered based on the mean velocity at the bifurcation. Further details on the velocity measurements are given in the Methods.
(EPS)

S2 Fig Relative difference (Rel. diff.) between consecutive RBC velocity measurements *in vivo*. (**A-B**) The relative difference between consecutive RBC velocity measurements is shown for all measurements at divergent (A) and convergent (B) bifurcations (Rel. diff. betw. measurements). The relative difference is computed with respect to the mean velocity of the capillary. Each value along the x-axis shows the three measurements per capillary. The different colours and symbols are used to discern different capillaries more easily. For illustrative purposes the measurements are ordered based on the largest relative difference for each capillary. The grey patch highlights all measurements where the maximum relative difference is < 10%. The dotted vertical line indicates where the first measurement differs by more than 10% from the mean velocity in the capillary. For 80% / 91% the difference between consecutive measurements is smaller than 10% at divergent/convergent bifurcations.
(EPS)

S3 Fig Velocity distribution for different simulation setups in the daughter vessels of divergent capillary bifurcations. (**A**) Velocity distribution for the simulation with red blood cells (RBCs) and with passive particles (pPs). The difference in the median for the simulation with RBCs and with pPs is to be expected because RBCs increase the overall flow resistance of the MVN and thus reduce the average velocity. (**B**) Velocity distribution for the simulation with RBCs and the simulation without phase separation. Here, in both simulations the impact of RBCs on the overall resistance is present and consequently no significant differences in the median is observed. We only depict velocities up to 2 mm/s. For the simulation with RBCs and without phase-separation this criterion includes 87% of all velocities in the daughter vessels of divergent bifurcations and 77% of all velocities for the simulation with pPs. In (A) and (B) the data from MVN 1 and MVN 2 is combined. Q1: lower quartile, Q3: upper quartile (Methods).
(EPS)

S4 Fig The two realistic microvascular networks (MVN) from the mouse parietal cortex. MVN 1 and MVN 2 are depicted in (A) and (B), respectively. The original networks have first been published in Blinder et al. [6].
(EPS)

S5 FigTime course of red blood cell velocities $v_{db}^{RBC,b_{i}}$ in the daughter vessels of six exemplary divergent capillary bifurcations from the *in vivo* measurements.We chose three examples of *well- balanced bifurcations* (**A, B, C**), two examples of *unbalanced bifurcations* (**D, E**) and one in the intermediate range (**F**). The definition of the different bifurcation types and of the relative velocity difference is given in the main text. The dotted lines show the median of the time course of each variable. Please note, that the velocities are recorded subsequently. The time on the x-axis is the time since the beginning of the measurement in Capillary 1 and 2, respectively. The general velocity heterogeneity becomes apparent as the median velocities range from 0.4–1.8 mm/s. $\left|\Delta^{r}v_{db}^{RBC}\right|$: Relative RBC velocity difference.(EPS)

S6 Fig**Time course of outflow velocities $v_{db}^{b_{i}}$ at three exemplary divergent capillary bifurcations for the simulation with red blood cells (RBCs) (left) and the simulation without phase separation (right).** Figs (**A, C, E**) show results for the simulation with RBCs and (**B, D, F**) for the simulation without phase separation. The first two examples (A-D) show bifurcations, which are not *well-balanced* in the simulation without phase separation but are *well-balanced* for the simulation with RBCs. In the third example (E-F) the bifurcation is *well-balanced* for the simulation with RBCs and for the simulation without phase separation. However, the relative velocity difference is smaller for the simulation with RBCs. (A) is also given in Fig 1E. Lower plot: instantaneous relative velocity difference $\Delta^{r}v_{db}$. The relative velocity difference is defined in the main text. The dotted lines show the median of the time course of each variable. The time courses have been smoothed (Methods).(EPS)

S7 Fig**Boxplot for the relative velocity difference (Rel. velocity dif., $\left|\Delta^{r}v_{db}\right|$) at *well-balanced* (A) and *unbalanced* (B) divergent capillary bifurcations for the simulation with red blood cells (with RBCs) and with passive particles (with pPs).** Only the subset of bifurcations that is *well-balanced*/ *unbalanced* in both simulation setups (with RBCs/ with pPs) is analysed. The underlying absolute velocity differences for *well-balanced* and *unbalanced* bifurcations is depicted in Fig 2A and 2B, respectively. The definition of the boxplots is given in the Methods. Statistical significance: Wilcoxon-signed-rank-test, ***: p-value < 0.001.(EPS)

S8 Fig Additional hematocrit distributions for the blood flow simulations. (**A**) Hematocrit distribution for all capillaries for the simulation with red blood cells (RBCs) and with passive particles (pPs). (**B**) Hematocrit distribution in the daughter vessels of divergent bifurcations for the simulation with RBCs and the simulation without phase separation (no phase-sep.). In (A) and (B) the data from MVN 1 and MVN 2 is combined. Q1: lower quartile, Q3: upper quartile. The definition of the boxplots is given in the Methods.
(EPS)

S9 Fig Simulated relative change in flow rate and in the number of red blood cells (nRBC) for different dilation factors. **(A-B)** Schematic of capillary dilation. (A) Exemplary divergent capillary bifurcation in the baseline case. D^baseline^: baseline capillary diameter. (B) Schematic how the vessel diameter of the capillary is changed. Note that the diameters at the divergent bifurcation remain constant and that only the second segment of the capillary is dilated. The length of the segment adjacent to the bifurcation (L^constant^) is set to six times the length of a red blood cell (RBC, Methods). D^dilated^: dilated capillary diameter. (**C-H**) Relative change in flow rate for a dilated segment of 100 μm (C-E) and in the number of RBCs, nRBC (F-H) for the mother and the two daughter vessels in response to capillary dilation. (C, F) Dilation factor: fDil = 1.1, (D, E) fDil = 1.05 and (E, H) fDil = 1.025. For each dilation factor, 25 different capillary dilations have been performed at *well-balanced bifurcations*. The changes increase for larger dilation factors. The definition of the boxplots is given in the Methods. Statistical significance: Wilcoxon-signed-rank-test, ***: p-value < 0.001.
(EPS)

S10 Fig Simulated relative change in flow rate and in the number of red blood cells (nRBC) for different simulation setups and different bifurcation types. Relative change in flow rate for a dilated segment of 100 μm (**A-D**) and in nRBC (**E-G**) for the mother and the two daughter vessels in response to capillary dilation by 10%. (A-B) and (E-F) show results for the simulation with red blood cells (RBCs) at *well-balanced* (A, E) and *unbalanced bifurcations* (B, F), respectively. (C) and (G) depict the relative changes at *well-balanced bifurcations* for the simulation without phase separation and (D) shows the relative change in flow rate for the simulation with passive particles (pPs). (A) and (E-G) are also depicted in Fig 3 and are only added to facilitate comparison. The definition of the boxplots is given in the Methods. Statistical significance: Wilcoxon-signed-rank-test, ***: p-value < 0.001, ns: non-significant.
(EPS)

S11 Fig Simulated relative flow change and change in the number of red blood cells (nRBC) as a function of the dilated vessel length. (**A**) Relative flow change and (**B**) relative change in nRBC over the dilated vessel length for all capillary dilations of 10%. The relative nRBC change does not depend on the dilated vessel length. The definition of *well-balanced* and *unbalanced bifurcations* is given in the main text.
(EPS)

S12 FigSensitivity analysis on the chosen threshold for *well-balanced* and *unbalanced bifurcations* for the simulated relative change in flow rate in response to a capillary dilation of 10%.(**A-B**) Simulated relative change in flow rate in the dilated vessel (Daughter 1 –dil.) in response to a capillary dilation of 10% along a 100 μm segment at *well-balanced* (A) and *unbalanced bifurcations* (B). (**C-D**) Simulated relative change in flow rate in the undilated vessel (Daughter 2 –const.) in response to a capillary dilation of 10% along a 100 μm segment at *well-balanced* (C) and *unbalanced bifurcations* (D). Each of the boxplots shows the results for a different threshold value for *well-balanced* ((1)-(3)) and *unbalanced bifurcations* ((4)-(6)), respectively. The legend for the thresholds is given on the right. Statistical significance: Mann-Whitney U Test, ***: p<0.001, ns: non-significant. Sample size for different thresholds: (1) n = 24, (2) n = 48, (3) n = 72, (4) n = 94, (5) n = 70, (6) n = 58. Definition boxplot: Methods (**E**) Raw data of the simulated relative change in flow rate in the dilated and the undilated vessel in response to a capillary dilation of 10% along a 100 μm segment for different relative velocity differences $\left|\Delta^{r}v_{db}\right|$. A weak negative correlation can be observed for the relative change in flow rate in the undilated vessel. The weak correlation shows why categorizing the bifurcations into *well-balanced* and *unbalanced bifurcations* facilitates the comparison of the simulation results.(EPS)

S13 FigSensitivity analysis on the chosen threshold for *well-balanced* and *unbalanced bifurcations* for the simulated relative change in the number of red blood cells (nRBC) in response to a capillary dilation of 10%.(**A-B**) Simulated relative change in nRBC in the dilated vessel (Daughter 1 –dil.) in response to a capillary dilation of 10% at *well-balanced* (A) and *unbalanced bifurcations* (B). (**C-D**) Simulated relative change in nRBC in the undilated vessel (Daughter 2 –const.) in response to a capillary dilation of 10% at *well-balanced* (C) and *unbalanced bifurcations* (D). Each of the boxplots shows the results for a different threshold value for *well-balanced* ((1)-(3)) and *unbalanced bifurcations* ((4)-(6)), respectively. The legend for the thresholds is given on the right. Statistical significance: Mann-Whitney U Test, ***: p<0.001, ns: non-significant. Sample size for different thresholds: (1) n = 24, (2) n = 48, (3) n = 72, (4) n = 94, (5) n = 70, (6) n = 58. Definition boxplot: Methods (**E**) Raw data of the simulated relative change in nRBC in the dilated and the undilated vessel in response to a capillary dilation of 10% for different relative velocity differences $\left|\Delta^{r}v_{db}\right|$. A weak negative correlation can be observed for the relative change in nRBC in the dilated vessel. For the relative change in nRBC in the undilated vessel the correlation is positive. The weak correlations show why categorizing the bifurcations into *well-balanced* and *unbalanced bifurcations* facilitates the comparison of the simulation results.(EPS)

S14 Fig Various characteristic measures on the distribution of *well-balanced bifurcations* (wb-bif.) in the realistic microvascular networks (MVN). (**A**) Relative number of *well-balanced bifurcations* per analysis layer (AL). (**B**) Minimum Euclidean distance between *well-balanced bifurcations*. (Dist. wb-bif.) (**C-D**) Minimum Euclidean distance between *well-balanced bifurcation* and descending arteriole, DA (C)/ ascending venule, AV (D). (**E**) Difference (Diff.) between the minimum path length from *well-balanced bifurcation* to AV and to DA. The minimum path length to DA and AV is depicted in Fig 5B. Each characteristic is averaged for the five ALs. The *well-balanced bifurcations* are associated with the AL based on their depth. Details on the computation of the different measures are provided in the Methods. The results in (B-E) have been tested for statistical significance. The p-values of the statistical tests are provided in S4–S6 Tables and S9 Table.
(EPS)

S1 Table Statistical analysis whether the average simulated relative changes in response to capillary dilation differ significantly from 0. (DOCX)

S2 Table Comparison of the results for capillary dilation at *well-balanced bifurcations* for the simulation with red blood cells (RBCs) with the other simulation setups. (DOCX)

S3 Table Statistical measures for the relative velocity differences at different bifurcation types for the *in vivo* experiments and the simulations (Fig 1F–1K). (DOCX)

S4 Table Statistical comparison (p-values) of the Euclidean distance between *well-balanced bifurcations* over cortical depth for microvascular network 1 (MVN 1) and MVN 2. (DOCX)

S5 Table Statistical comparison (p-values) of the minimum Euclidean distance between well-balanced bifurcations and descending arteriole (DA) over cortical depth for microvascular network 1 (MVN 1) and MVN 2. (DOCX)

S6 Table Statistical comparison (p-values) of the minimum Euclidean distance between *well-balanced bifurcations* and ascending venule (AV) over cortical depth for microvascular network 1 (MVN 1) and MVN 2. (DOCX)

S7 Table Statistical comparison (p-values) of the minimum path length between *well-balanced bifurcations* and descending arteriole (DA) over cortical depth for microvascular network 1 (MVN 1) and MVN 2. (DOCX)

S8 Table Statistical comparison (p-values) of the minimum path length between *well-balanced bifurcations* and ascending venule (AV) over cortical depth for microvascular network 1 (MVN 1) and MVN 2. (DOCX)

S9 Table Statistical comparison (p-values) of the difference between the minimum path length between *well-balanced bifurcations* and descending arteriole (DA) and ascending venule (AV) over cortical depth for microvascular network 1 (MVN 1) and MVN2. (DOCX)
