# Supplementary material for: Red blood cells stabilize flow in brain microvascular networks
Source: PLoS Comput Biol. 2019 Aug 30;15(8):e1007231. doi: 10.1371/journal.pcbi.1007231 (PMC6750893; doi:10.1371/journal.pcbi.1007231)
Supplement: S1 Table — (DOCX) [file pcbi.1007231.s015.docx]

**S1 Table**. Statistical analysis whether the average simulated relative changes in response to capillary dilation differ significantly from 0.

|  |  | **p-value: Relative flow change** | | |  | **p-value: Relative nRBC change** | | |
| --- | --- | --- | --- | --- | --- | --- | --- | --- |
|  |  | Daughter 1 - dilated | Mother - constant | Daughter 2 - constant |  | Daughter 1 - dilated | Mother - constant | Daughter 2 - constant |
|  |  |  |  |  |  |  |  |  |
| ***Well-balanced*** |  |  |  |  |  |  |  |  |
| with RBCs |  | 7.87e^-18^ | 2.61e^-15^ | 0.451 (ns) |  | 8.15e^-16^ | 0.273 (ns) | 1.80e^-07^ |
| no phase separation |  | 4.27e^-21^ | 9.42e^-18^ | 3.17e^-09^ |  | 8.49e^-27^ | 0.022 (ns) | 0.082 (ns) |
|  |  |  |  |  |  |  |  |  |
| ***Unbalanced*** |  |  |  |  |  |  |  |  |
| with RBCs |  | 1.76e^-19^ | 1.09e^-12^ | 1.54e^-08^ |  | 1.43e^-19^ | 0.233 (ns) | 0.028 (ns) |

p-value for the two-sided t-test. Relative changes with a p-value < 0.001 are considered significant. ns: non-significant (p > 0.001). The sample size is 50 and 70 for *well-balanced* and *unbalanced bifurcations*, respectively. The underlying data is given in the boxplots in S10 Fig.
