# Supplementary material for: Red blood cells stabilize flow in brain microvascular networks
Source: PLoS Comput Biol. 2019 Aug 30;15(8):e1007231. doi: 10.1371/journal.pcbi.1007231 (PMC6750893; doi:10.1371/journal.pcbi.1007231)
Supplement: S2 Table — (DOCX) [file pcbi.1007231.s016.docx]

**S2 Table.** Comparison of the results for capillary dilation at *well-balanced bifurcations* for the simulation with red blood cells (RBCs) with the other simulation setups.

|  |  | **p-value: Relative flow change** | | |  | **p-value: Relative nRBC change** | | |
| --- | --- | --- | --- | --- | --- | --- | --- | --- |
|  |  | Daughter 1 - dilated | Mother - constant | Daughter 2 - constant |  | Daughter 1 - dilated | Mother - constant | Daughter 2 - constant |
|  |  |  |  |  |  |  |  |  |
| With RBCs -  *Unbalanced* |  | 0.104 * (ns) | - | 3.43e^-08^ † |  | 3.15e^-10^ † | - | 3.10e^-12^ * |
|  |  |  |  |  |  |  |  |  |
| No phase separation – *well-balanced* |  | 3.98e^-04^ * | - | 9.53e^-08^ † |  | 3.67e^-13^ † | - | 1.11e^-11^ * |

p-value of the one sided Mann-Whitney U Test. The symbols show which alternative hypothesis has been used. *: The average relative change is smaller at *well-balanced bifurcations*. †: The average relative change is larger at *well-balanced bifurcations*. A p-value < 0.001 is considered to be significant. Non-significant differences are marked with (ns). Note that the sign of the average relative flow change matters, e.g. for the relative flow change in daughter vessel 2 the average at *well-balanced bifurcations* is close to 0, while it is < 0 for the other setups. Consequently, our alternative hypothesis is that the relative flow change in daughter vessel 2 is greater than in the other setups. The sample size is 50 and 70 for *well-balanced* and *unbalanced bifurcations*, respectively. RBCs: Red blood cells. nRBC: number of RBCs.
