# Supplementary material for: Red blood cells stabilize flow in brain microvascular networks
Source: PLoS Comput Biol. 2019 Aug 30;15(8):e1007231. doi: 10.1371/journal.pcbi.1007231 (PMC6750893; doi:10.1371/journal.pcbi.1007231)
Supplement: S3 Table — (DOCX) [file pcbi.1007231.s017.docx]

**S3 Table.** Statistical measures for the relative velocity differences at different

bifurcation types for the *in vivo* experiments and the simulations (Figs 1F-K).

|  |  | **Mean** | **Median** | **Std** | **SEM** | **Q1** | **Q3** | **Skew** | **DoWB** | **n** |
| --- | --- | --- | --- | --- | --- | --- | --- | --- | --- | --- |
|  |  |  |  |  |  |  |  |  |  |  |
| ***In vivo*** |  |  |  |  |  |  |  |  |  |  |
| Divergent |  | 0.381 | 0.269 | 0.312 | 0.051 | 0.116 | 0.584 | 0.839 | 0.395 | 38 |
| Convergent |  | 0.496 | 0.479 | 0.278 | 0.049 | 0.291 | 0.635 | 0.500 | 0.156 | 32 |
|  |  |  |  |  |  |  |  |  |  |  |
| **Simulation with RBCs** |  |  |  |  |  |  |  |  |  |  |
| Divergent |  | 0.685 | 0.556 | 0.549 | 0.006 | 0.188 | 1.105 | 0.551 | 0.261 | 7285 |
| Convergent |  | 0.835 | 0.783 | 0.535 | 0.007 | 0.371 | 1.264 | 0.256 | 0.136 | 6541 |
|  |  |  |  |  |  |  |  |  |  |  |
| **Simulation with pPs** |  |  |  |  |  |  |  |  |  |  |
| Divergent |  | 0.823 | 0.739 | 0.559 | 0.007 | 0.344 | 1.258 | 0.402 | 0.152 | 7262 |

Std: Standard deviation. SEM: Standard error of the mean. Q1/Q3: first/third quartile. Skew: Skewness. DoWB: Degree of *well-balanced bifurcations*, i.e. the ratio of the number of *well-balanced* divergent/convergent bifurcations to the total number of divergent/convergent bifurcations. n: The total number of divergent/convergent bifurcations. RBCs: Red blood cells. pPs: passive particles (Methods).
