# Supplementary material for: Red blood cells stabilize flow in brain microvascular networks
Source: PLoS Comput Biol. 2019 Aug 30;15(8):e1007231. doi: 10.1371/journal.pcbi.1007231 (PMC6750893; doi:10.1371/journal.pcbi.1007231)
Supplement: S4 Table — (DOCX) [file pcbi.1007231.s018.docx]

**S4 Table.** Statistical comparison (p-values) of the Euclidean distance between *well-balanced bifurcations* over cortical depth for microvascular network 1 (MVN 1) and MVN 2.

|  | **AL1** | **AL2** | **AL3** | **AL4** | **AL5** |
| --- | --- | --- | --- | --- | --- |
| **AL1** |  | 3.34e^-06^ | 0.005 | 8.52e^-04^ | 0.033 |
| **AL2** | 4.42e^-04^ |  | 0.022 | 0.210 | 0.030 |
| **AL3** | 0.019 | 0.093 |  | 0.187 | 0.359 |
| **AL4** | 0.131 | 0.008 | 0.115 |  | 0.137 |
| **AL5** | 0.192 | 5.42e^-05^ | 0.002 | 0.030 |  |

To compare differences over cortical depth all analysis layers (AL) are compared with each other. The results for MVN 1 are depicted in the upper right part of the table and for MVN 2 in the lower left. The Mann-Whitney U Test is used to test for statistical significance. A p-value < 0.001 is considered as significant. Significant results are highlighted in red. The approach to compute the Euclidean distance between *well-balanced bifurcations* is described in the Methods. The median values of the underlying distributions are depicted in S14 Fig B.
