# Supplementary material for: Red blood cells stabilize flow in brain microvascular networks
Source: PLoS Comput Biol. 2019 Aug 30;15(8):e1007231. doi: 10.1371/journal.pcbi.1007231 (PMC6750893; doi:10.1371/journal.pcbi.1007231)
Supplement: S5 Table — (DOCX) [file pcbi.1007231.s019.docx]

**S5 Table.** Statistical comparison (p-values) of the minimum Euclidean distance between *well-balanced bifurcations* and descending arteriole (DA) over cortical depth for microvascular network 1 (MVN 1) and MVN 2.

|  | **AL1** | **AL2** | **AL3** | **AL4** | **AL5** |
| --- | --- | --- | --- | --- | --- |
| **AL1** |  | 6.13e^-04^ | 8.43e^-05^ | 0.002 | 0.036 |
| **AL2** | 9.78e^-17^ |  | 0.344 | 0.460 | 0.090 |
| **AL3** | 1.84e^-12^ | 0.049 |  | 0.352 | 0.044 |
| **AL4** | 2.38e^-05^ | 1.03e^-05^ | 0.004 |  | 0.106 |
| **AL5** | 0.006 | 2.70e^-06^ | 4.23e^-04^ | 0.150 |  |

To compare differences over cortical depth all analysis layers (AL) are compared with each other. The results for MVN 1 are depicted in the upper right part of the table and for MVN 2 in the lower left. The Mann-Whitney U Test is used to test for statistical significance. A p-value < 0.001 is considered as significant. Significant results are highlighted in red. The approach to compute the Euclidean distance between *well-balanced bifurcation* and DA is described in the Methods. The median values of the underlying distributions are depicted in S14 Fig C.
