# Supplementary material for: Red blood cells stabilize flow in brain microvascular networks
Source: PLoS Comput Biol. 2019 Aug 30;15(8):e1007231. doi: 10.1371/journal.pcbi.1007231 (PMC6750893; doi:10.1371/journal.pcbi.1007231)
Supplement: S6 Table — (DOCX) [file pcbi.1007231.s020.docx]

**S6 Table.** Statistical comparison (p-values) of the minimum Euclidean distance between *well-balanced bifurcations* and ascending venule (AV) over cortical depth for microvascular network 1 (MVN 1) and MVN 2.

|  | **AL1** | **AL2** | **AL3** | **AL4** | **AL5** |
| --- | --- | --- | --- | --- | --- |
| **AL1** |  | 4.37e^-06^ | 1.07e^-09^ | 3.16e^-07^ | 8.54e^-19^ |
| **AL2** | 9.79e^-14^ |  | 0.004 | 0.076 | 1.56e^-11^ |
| **AL3** | 8.81e^-19^ | 0.015 |  | 0.240 | 1.71e^-05^ |
| **AL4** | 2.51e^-11^ | 0.324 | 0.097 |  | 2.35e^-06^ |
| **AL5** | 5.57e^-24^ | 3.13e^-09^ | 4.90e^-06^ | 2.36e^-07^ |  |

To compare differences over cortical depth all analysis layers (AL) are compared with each other. The results for MVN 1 are depicted in the upper right part of the table and for MVN 2 in the lower left. The Mann-Whitney U Test is used to test for statistical significance. A p-value < 0.001 is considered as significant. Significant results are highlighted in red. The approach to compute the Euclidean distance *well-balanced bifurcation* and AV is described in the Methods. The median values of the underlying distributions are depicted in S14 Fig D.
