# Supplementary material for: Red blood cells stabilize flow in brain microvascular networks
Source: PLoS Comput Biol. 2019 Aug 30;15(8):e1007231. doi: 10.1371/journal.pcbi.1007231 (PMC6750893; doi:10.1371/journal.pcbi.1007231)
Supplement: S7 Table — (DOCX) [file pcbi.1007231.s021.docx]

**S7 Table.** Statistical comparison (p-values) of the minimum path length between *well-balanced bifurcations* and descending arteriole (DA) over cortical depth for microvascular network 1 (MVN 1) and MVN 2.

|  | **AL1** | **AL2** | **AL3** | **AL4** | **AL5** |
| --- | --- | --- | --- | --- | --- |
| **AL1** |  | 9.66^-06^ | 0.001 | 3.98e^-08^ | 0.377 |
| **AL2** | 1.31e^-09^ |  | 0.223 | 0.041 | 6.87e^-04^ |
| **AL3** | 5.91e^-09^ | 0.498 |  | 0.011 | 0.131 |
| **AL4** | 0.014 | 1.53e^-04^ | 2.82e^-04^ |  | 5.83e^-05^ |
| **AL5** | 0.189 | 4.30e^-05^ | 1.37e^-04^ | 0.192 |  |

To compare differences over cortical depth all analysis layers (AL) are compared with each other. The results for MVN 1 are depicted in the upper right part of the table and for MVN 2 in the lower left. The Mann-Whitney U Test is used to test for statistical significance. A p-value < 0.001 is considered as significant. Significant results are highlighted in red. The approach to compute the minimum path length between *well-balanced bifurcation* and DA is described in the Methods. The median values of the underlying distributions are depicted in Fig 5B.
