# Supplementary material for: Red blood cells stabilize flow in brain microvascular networks
Source: PLoS Comput Biol. 2019 Aug 30;15(8):e1007231. doi: 10.1371/journal.pcbi.1007231 (PMC6750893; doi:10.1371/journal.pcbi.1007231)
Supplement: S8 Table — (DOCX) [file pcbi.1007231.s022.docx]

**S8 Table.** Statistical comparison (p-values) of the minimum path length between *well-balanced bifurcations* and ascending venule (AV) over cortical depth for microvascular network 1 (MVN 1) and MVN 2.

|  | **AL1** | **AL2** | **AL3** | **AL4** | **AL5** |
| --- | --- | --- | --- | --- | --- |
| **AL1** |  | 0.001 | 1.25e^-04^ | 1.81e^-04^ | 1.43e^-10^ |
| **AL2** | 5.21e^-11^ |  | 0.169 | 0.090 | 1.16e^-06^ |
| **AL3** | 2.84e^-07^ | 0.063 |  | 0.400 | 1.06e^-04^ |
| **AL4** | 3.80e^-06^ | 0.237 | 0.348 |  | 7.98e^-04^ |
| **AL5** | 6.12e^-08^ | 0.062 | 0.009 | 0.034 |  |

To compare differences over cortical depth all analysis layers (AL) are compared with each other. The results for MVN 1 are depicted in the upper right part of the table and for MVN 2 in the lower left. The Mann-Whitney U Test is used to test for statistical significance. A p-value < 0.001 is considered as significant. Significant results are highlighted in red. The approach to compute the minimum path length between *well-balanced bifurcation* and AV is described in the Methods. The median values of the underlying distributions are depicted in Fig 5B.
