# Supplementary material for: Red blood cells stabilize flow in brain microvascular networks
Source: PLoS Comput Biol. 2019 Aug 30;15(8):e1007231. doi: 10.1371/journal.pcbi.1007231 (PMC6750893; doi:10.1371/journal.pcbi.1007231)
Supplement: S9 Table — (DOCX) [file pcbi.1007231.s023.docx]

**S9 Table.** Statistical comparison (p-values) of the difference between the minimum path length between *well-balanced bifurcations* and descending arteriole (DA) and ascending venule (AV) over cortical depth for microvascular network 1 (MVN 1) and MVN2.

|  | **AL1** | **AL2** | **AL3** | **AL4** | **AL5** |
| --- | --- | --- | --- | --- | --- |
| **AL1** |  | 1.36e^-06^ | 1.49e^-06^ | 2.38e^-09^ | 1.93e^-06^ |
| **AL2** | 1.52e^-15^ |  | 0.258 | 0.012 | 0.032 |
| **AL3** | 5.16e^-13^ | 0.211 |  | 0.074 | 0.074 |
| **AL4** | 5.22e^-06^ | 0.014 | 0.073 |  | 0.401 |
| **AL5** | 1.90e^-06^ | 0.437 | 0.364 | 0.101 |  |

To compare differences over cortical depth all analysis layers (AL) are compared with each other. The results for MVN 1 are depicted in the upper right part of the table and for MVN2 in the lower left part. The p-value of the Mann-Whitney U Test is used to test for statistical significance. A p-value < 0.001 is considered to be significant. Significant results are highlighted in red. The approach to compute the compute the minimum path length is described in the Methods. The median values of the underlying distributions are depicted in S14 Fig E.
